# Tracking Gold Nanorods’ Interaction with Large 3D Pancreatic-Stromal Tumor Spheroids by Multimodal Imaging: Fluorescence, Photoacoustic, and Photothermal Microscopies

**DOI:** 10.1038/s41598-020-59226-6

**Published:** 2020-02-25

**Authors:** Emilie Darrigues, Zeid A. Nima, Dmitry A. Nedosekin, Fumiya Watanabe, Karrer M. Alghazali, Vladimir P. Zharov, Alexandru S. Biris

**Affiliations:** 1 Center for Integrative Nanotechnology Sciences, University of Arkansas at Little Rock, 2801 S University Avenue, Little Rock, AR 72204 USA; 2 Arkansas Nanomedicine Center, University of Arkansas for Medical Sciences, 4301 West Markham Street, Little Rock, AR 72205 USA

**Keywords:** Cancer microenvironment, Nanoparticles

## Abstract

Pancreatic cancer is one of the most complex types of cancers to detect, diagnose, and treat. However, the field of nanomedicine has strong potential to address such challenges. When evaluating the diffusion and penetration of theranostic nanoparticles, the extracellular matrix (ECM) is of crucial importance because it acts as a barrier to the tumor microenvironment. In the present study, the penetration of functionalized, fluorescent gold nanorods into large (>500 μm) multicellular 3D tissue spheroids was studied using a multimodal imaging approach. The spheroids were generated by co-culturing pancreatic cancer cells and pancreatic stellate cells in multiple ratios to mimic variable tumor-stromal compositions and to investigate nanoparticle penetration. Fluorescence live imaging, photothermal, and photoacoustic analysis were utilized to examine nanoparticle behavior in the spheroids. Uniquely, the nanorods are intrinsically photoacoustic and photothermal, enabling multi-imaging detection even when fluorescence tracking is not possible or ideal.

## Introduction

Nanomedicine for cancer treatments—the use of nanosized structures in cancer therapeutics and/or imaging—has been heavily investigated over the last two decades^1^. Nano-sized delivery systems are useful theragnostic agents for enhanced tumor penetration, accumulation, and targeting potency. Studies of nanoparticle (NP)-based therapies have mainly focused on targeted cancer cell treatment but also include cancer stem cells^2^, stromal cancer microenvironment^3–5^, and/or cellular immune system^6,7^. As a physical barrier that limits NP penetration and distribution, the extracellular matrix (ECM) is a crucial concern when evaluating the effects of nanoscale cancer therapies. Recent work showed that the ECM can be regulated by using gold NPs to interrupt the crosstalk between cancer cells and stellate cells, which reeducates the stellate cells^8,9^.

However, the development of nanosystems for cancer treatment has been hampered by the limitations of current *in vitro* models, which are generally based on two-dimensional (2D) cell cultures. These 2D models struggle to provide an accurate representation of the *in vivo* environment and its components, which include the dynamic tumor microenvironment (TME), cell heterogeneity, and nutrient and pH gradient interaction between cells and the ECM^10^. The real TME is composed not only of cancer cells but also of others cells such as fibroblasts, cancer-associated fibroblasts (CAFs), stromal cells, myofibroblasts, endothelial cells, adipocytes, various immune cells, and extra-abundant compromised ECM. These heterocellular components can induce adaptive survival mechanisms of cancer such as treatment resistance, a leading cause of cancer-related mortality and one of the greatest challenges in cancer treatment^11^. The cellular responses and cell signaling that take place in the TME often cannot be mimicked in a 2D *in vitro* model.

The limitations of 2D models have affected the development of nanomedicine approaches to treat pancreatic cancer^12^, particularly pancreatic ductal adenocarcinoma (PDAC)^13^. PDAC is composed of cancer cells, as well as pancreatic stellate cells (PSCs) differentiated into CAFs^13^. These cells, which are similar to myofibroblasts and originate in exocrine areas of the pancreas, are homeostatic, quiescent, lipid-storing cells. When activated by pro-inflammatory cancer signaling, these cells may proliferate, migrate, and accumulate in the tumor by secreting a considerable amount of ECM, which develops a protective, growth-permissive fibrotic stroma barrier. Activated PSCs and CAFs are highly modulated by paracrine/autocrine cell signaling and are involved in the full remodeling of the TME, inducing hypoxia and pH variation^14–17^. Consequently, CAFs influence PDAC disease progression, tumorigenesis, metastasis, immune reaction, therapeutic response, drug resistance, and treatment failure^18–21^. Multicellular, 3D pancreatic tumor models co-cultured with CAFs have been shown to simulate abundant desmoplasia with ECM. For example, a 3D PDAC *in vitro* model, made by co-culturing pancreatic cancer cells and stellate cells, generated a rich, dense, active, and mechanically stable stromal compartment^22,23^, relatively similar to that of real tumors seen in biopsied tissues. Based on these results, the 3D *in vitro* culture model could be highly useful for PDAC-oriented nanomedicine screening^10,24–26^.

Nanoparticles can have specific light interaction characteristics, making them suitable for multimodal detection, such as Raman spectroscopy^27–29^, photoacoustic analysis^30^, magnetic resonance imaging^31^, two-photon microscopy^32^, or even a combination of these methods^33^, in 2D, 3D and *in-vivo* models^34^. However, few of these techniques have been applied in 3D cancer models (spheroids or phantoms)^35^. Imaging-guided NP tracking is a major medical need and research focus, as it would enable drug release to be tracked and mapped in real-time^36^. Recently, a doxorubicin-loaded polymeric NP diffusion was tracked via confocal scanning microscopy in a complex pancreatic cancer 3D model made of cancer, endothelial, and fibroblast cells. Results showed that confocal scanning microscopy is limited in its ability to provide accurate deep penetration (>100 μm) data on the diffusion of small molecules due to the progressive loss of their fluorescence signal^37^. This problem may be addressed by utilizing different characterization instruments based on the specific light-matter properties needed.

Additionally, drug-loaded nanocarriers can be tailored to be responsive to stimuli such as temperature, pH, light, magnetic stimulation, and enzymatic processes, which enhances their potential as highly functional drug delivery systems^38–40^. For example, a series of chemical conjugations (EDC/NHS (1-Ethyl-3-(3-dimethylaminopropyl)carbodiimide/ N-hydroxysuccinimide) reaction, protonation/deprotonation balance)was used to cause a pH-responsive bond, enabling pH-triggered controlled drug release from the nanosystem into the tumor tissue^41,42^. Because 3D cancer models (especially those composed of stromal cells and ECM components) are designed to mimic the complex *in vivo* environment with low pH—induced by hypoxic conditions—drugs can detach off the surface of nanocarriers before accessing cells. Similarly, fluorescent dyes could detach from nanocarriers in certain conditions, which makes tracking based on fluorescence signals less reliable. Despite these possible shortcomings, 3D models are still excellent tools for gaining insight on how multifunctional nanocarriers interact with *in vivo* systems.

**Figure 1:**
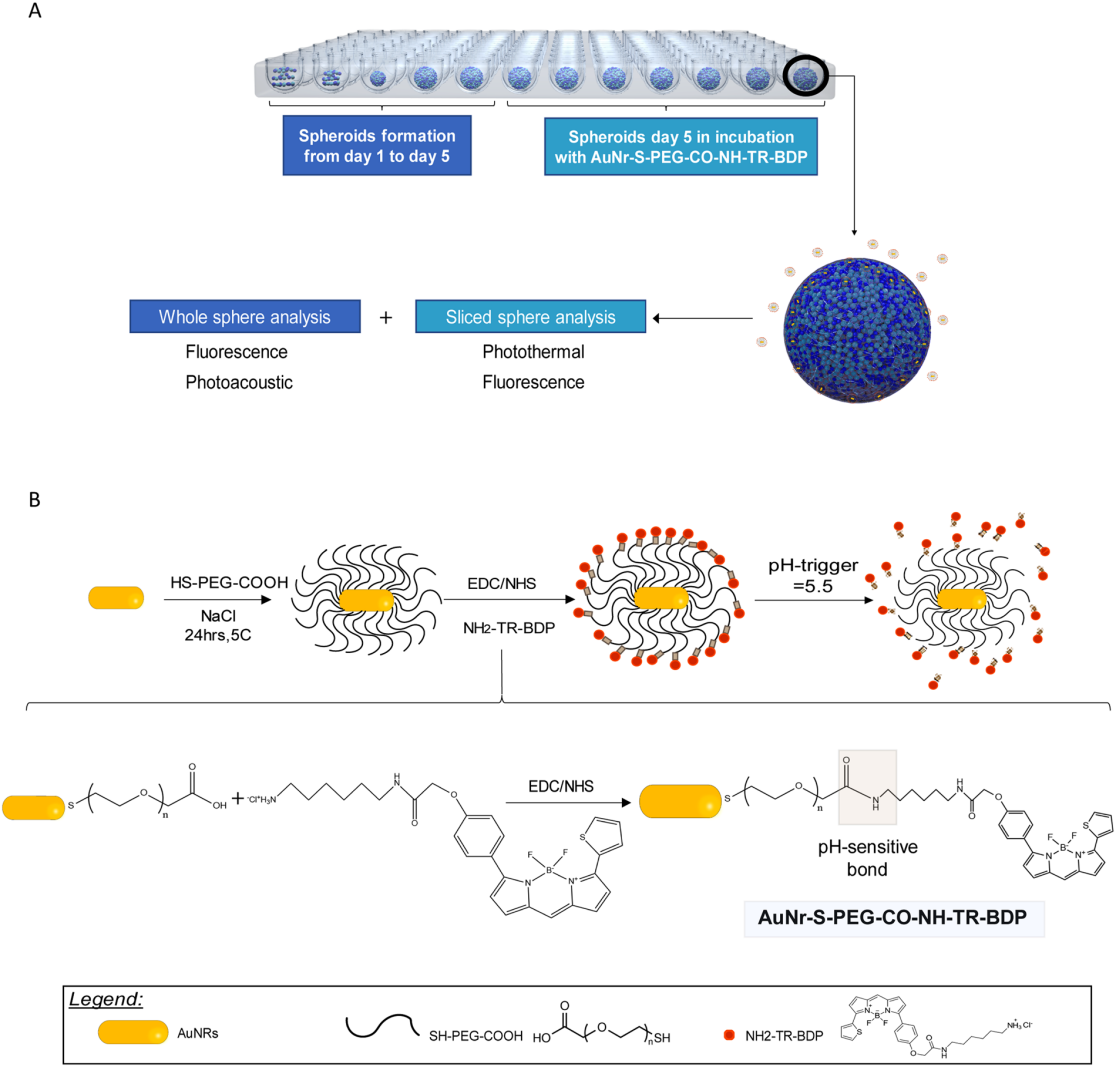
Schematic of the whole experimentation and of the AuNRs. (**A**) Schematic of the experimentation: spheroid formation (day 1 to day 5) in ultra-low-attachment 96-well plate, functionalized AuNR incubation, followed by characterization of the whole sphere and a section of it. (**B**) Schematic of AuNR functionalization from bare to covered with HS-PEG-COOH to allow EDC/NHS reaction conjugation of the fluorescent dye NH_2_-TR-BDP, which can be released by acid pH-trigger at a pH of 5.5.

Previously, we successfully used gold nanoparticles’ unique photoacoustic (PA) and photothermal (PT) signatures to study their theragnostic behavior and mechanisms of interactions with cells in 2D culture and *in vivo* cancer and immune system models^43–48^. Herein, we have developed an avascular 3D pancreatic microtumor model by varying the ratio of Panc-1/PSCs. These spheroids were incubated with gold nanorods (AuNRs, aspect ratio ~3), which had been functionalized with a biocompatible thiolated-polyethylene (HS-PEG-COOH) and conjugated with a fluorescent dye (BOPIDY type, NH_2_-TR-BDP). The first goal of this study was to confirm the ability of our pH-triggered gold nanoparticles to interact with a complex and large (>500 μm) 3D pancreatic model. Fluorescence and PA microscopy analyses of the whole spheroids were used to investigate the spheroids’ interaction with the NPs and determine the effect of the stroma on NP distribution within the complex spheroid multicellular environement. The second goal of the study was to learn if the data collected for the whole sphere and for the spheroid slices had similar features and, for the first time to the best of our knowledge, to compare fluorescence (from tag) vs. photothermal signatures (unique to the NP) (Fig. 1A). We utilized the unique, intrinsic PA and PT contrast signals of the NPs to identify them in the spheroids without relying only on fluorescent tagging, which enabled multi-imaging analysis and confirmed that tracked fluorescence signals might not always be related to the NP signal.

## Results

### Synthesis and characterization of nanoparticle conjugates

**Figure 2:**
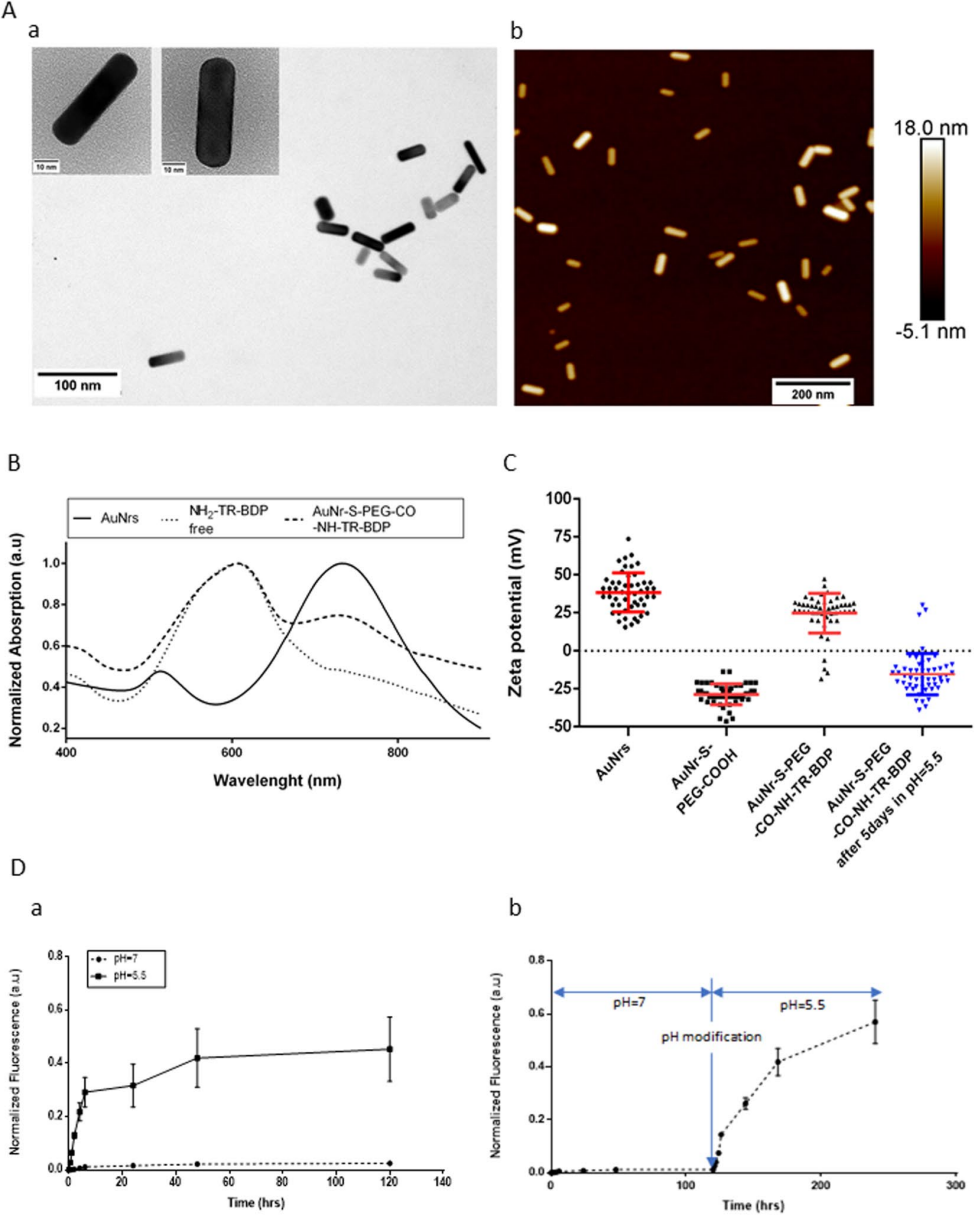
Characterization and analysis of the functionalized AuNRs. (**A**) (a) High-resolution TEM and (b) AFM images of bare gold nanorods (AuNRs). (**B**) UV-visible absorption curve of AuNRs, unconjugated NH_2_-TR-BDP, and fully conjugated AuNR-S-PEG-CO-NH-TR-BDP. (**C**) Zeta potential measured on AuNRs, AuNR-S-PEG-COOH, AuNR-S-PEG-CO-NH-TR-BDP, and AuNR-S-PEG-CO-NH-TR-BDP after 5 days of exposure to pH = 5.5. (**D**) (a) Normalized fluorescence release intensity of AuNR-S-PEG-CO-NH-TR-BDP incubated at 37 °C at 5%CO_2_ in pH = 7 and pH = 5.5 for 0.5hrs, 1 hr, 2 hrs, 4 hrs, 6 hrs, 24 hrs, 48 hrs, and 5 days, and, b) Normalized fluorescence release of nanoparticles incubated at pH = 7 for 5 days then incubated at pH = 5.5 for an additional 30 min, 1 hr, 2 hrs, 4 hrs, 6 hrs, 24 hrs, 48 hrs, and 5 days. This data is a compilation of 3 independent experiments; n = 3 per group, graph with SEM representation.

Gold nanorods (AuNR, majoritarily with an aspect ratio of length/diameter: ~3) were synthesized using a previously reported seed‐mediated method^45,49^. Performance, toxicity, and interaction of these AuNRs, functionalized with various substances, with cancer cells have already been analyzed in our previous studies^47,48^. In this work, fluorescent nano‐agents (nanorod conjugates) were prepared according to the EDC/NHS coupling reaction using both carboxylic (from HS-PEG-COOH) and amine (from BODIPY-TR-NH_2_) functional groups in a one‐step approach (Fig. 1B)^42^. The morphology and size of the gold nanorods were determined using transmission electron microscopy (TEM) and atomic force microscopy (AFM) (Fig. 2A). TEM images indicated that the majority of the nanorods had a length of ~35.0 nm  and a width of ~12.0 nm^45,46^. UV-visible spectra analysis confirmed the attachment of NH_2_-TR-BDP on the AuNR-S-PEG-COOH, forming AUNR-S-PEH-CO-NH-TR-BDP. The nanosystem showed two peaks at ~605 nm and ~740 nm, corresponding with the maximum wavelength absorption of free NH_2_-TR-BDP and the second peak of AuNR (the absorption of the longest dimension of the nanostructure), respectively (Fig. 2B).

Zeta potential was used to verify the UV-visible absorption spectra results by measuring the variation of the surface charge/potential during the different steps of conjugation. The values were ζ_AuNRs_ = 38 mV ± 2 mV, ζ_AuNR-S-PEG-COOH_ = −28 mV ± 1 mV, and ζ_AuNR-S-PEG-CO-NH-TR-BDP_ = 25 mV ± 2 mV, confirming the conjugation of the fluorescent dye to the nanoparticles (Fig. 2C). The AuNRs’ positive potential resulted from their dispersion in CTAB, a surfactant used during synthesis to make them shelf-stable in certain concentrations without sedimentation or structural modification. When the AuNRs were purified and covered by the negatively charged HS-PEG-COOH, their zeta potential became negative, confirming that the conjugation had been successful. However, after the EDC/NHS reaction, the potential of the functionalized fluorescent nanoparticles was raised, becoming positive. Within the structure of NH_2_-TR-BDP, NH_2_^+^ is a molecular functional group that can add a positive charge on the surface of the AuNRs. Thus, the nanosystem’s positive potential indicates that the NPs were functionalized not exclusively by EDC/NHS covalent bonding but possibly also by other interactions such as electrostatic and/or Π−Π stacking.

Fluorescence activation release was evaluated by pH trigger variation at pH = 7 using 1x PBS buffer and at pH = 5.5 using sodium acetate buffer (Fig. 2Da), with the cells being tracked at 0 hrs, 0.5 hrs, 1 hr, 2 hrs, 4 hrs, 6 hrs, 24 hrs, 48 hrs, and 5 days. Each point measured the same solution which had previously been dispersed by a sonicator probe in a fresh buffer before incubation at 37 °C. The early sign of release was measured after 1 hour, and the inflection point of the fluorescence release was identified after 6 hours. In the pH = 7 solution, after 5 days, a small amount of fluorescent dye had been released (Fig. S1), extremely insignificant compared to pH = 5.5, whose release was x10 times higher for the same time period. At the 5-day point, additional zeta potential measurements of the AuNRs incubated at pH = 5.5 were performed. The result for ζ_AuNR-S-PEG-CO-NH-TR-BDP_ (−15 mV ± 2 mV) (Fig. 2C) also confirmed the release of the dye, with a shift from positive to negative overall charge.

Next, the conjugated AuNRs that had been incubated at pH = 7 were incubated at pH = 5.5 for 5days. (Fig. 2D,b) These AuNRs produced a fluorescence release after the first minutes of exposure to the acidic buffer. The fluorescence profile confirmed the stability of the conjugated AuNRs at pH = 7 and only a possible significant release at pH = 5.5, additionally showing that temperature and sonication do not seem to influence the dye release. It was also confirmed that the fluorescence detected in the spheroids could only have originated from the conjugated AuNRs, not from any free dye remaining in the solution or prematurely released before interaction with the cells or spheroid microenvironment. Considering the fast dye release at low pH values, other biochemical reactions/processes (as previously described enzymatic degradation of the disulfide bond PEG-S-S-AuNRs^50,51^), the kinetics of fluorescence release inside the cells is expected to occur relatively fast.

### Generation and characterization of 3D spheroids

Before investigating the nanoparticles’ interaction with the spheroids, we characterized the spheroids. All spheroids were made from cells cultured in 2D on individually cultured Panc-1 (passage ~4 to 12) and PSCs (passage ~3 to 7). The initial total cell seeding number was fixed at ~10,000 cells/well. The only varied parameter was the co-culture cell ratio of pancreatic cancer to stellate cells, with Panc-1-to-PSCs ratios of 5-1, 1-1, and 1–2. The ratios were determined based on published studies on 3D pancreatic cancer cells made with stellate cells or various fibroblasts^25,52^. The 1–2 ratio of Panc-1/PSCs was chosen to be in the maximal hypoxic condition.

**Figure 3:**
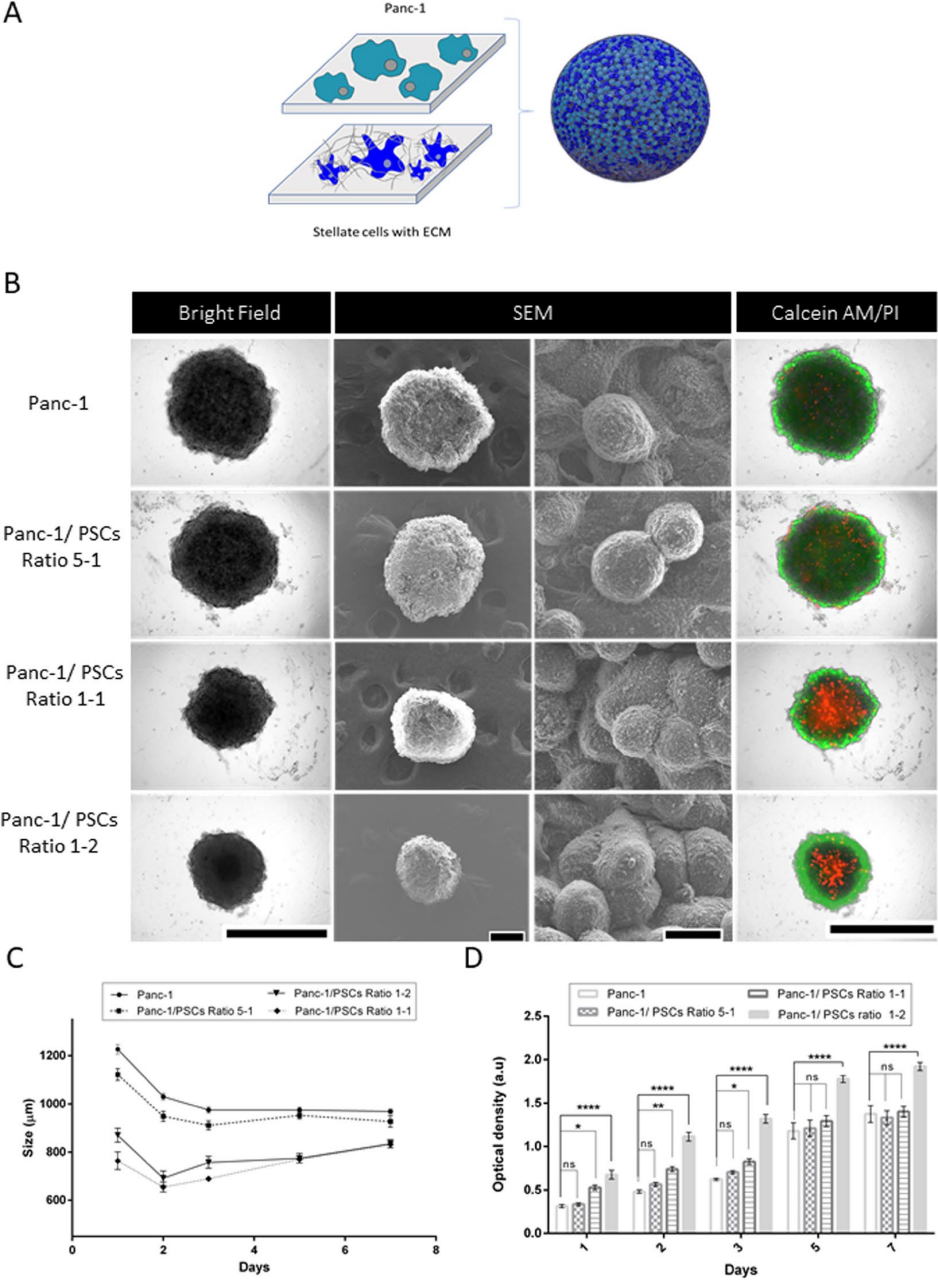
Characterization of the spheroids incubated in a 96-well, ultra-low adhesion plate. (**A**) Schematic of 3D culture formation from 2D cell culture of Panc-1 and stellate cells. (**B**) Images of spheroids, from left to right: optical images in the BF (scale bar: 1000 μm), SEM images at magnification x100 (scale bar: 200 μm) and x3000 (scale bar: 10 μm), and fluorescence optical overlap BF imaging of the proliferation and viability of 3D culture with calcein AM (green)/ propidium iodide (PI, red) (scale bar: 1000 μm). (**C**) Evolution of the size and (**D**) optical density (determined using ImageJ NIH software) of the spheroids at different stellate cell ratios in function of days of formation, from day 1 to day 7 (n = 20 in C and D, and p < 0.005 as determined by two-way ANOVA using Sidak correction in D).

We performed optical bright field (BF) (Cytation 5) and high resolution scanning electron microscopy (SEM) imaging to monitor the spheroids’ growth profile, including real-time size monitoring, optical density analysis, and viability analysis (Fig. 3A–D). Microscopic observation was performed as preliminary data to confirm the sphere-like shape of the spheroids. The sphericity index was investigated by comparing opposite diameter measurements for the same sphere after 5 days. The measurements were relatively similar for all the spheroids, verifying their spherical properties (Panc-1: 0.93 ± 0.02, Panc-1-PSC ratio 5-1: 0.92 ± 0.92, Panc-1-PSC ratio 1-1: 0.96 ± 0.01, Panc-1-PSC ratio 1-2: 0.85 ± 0.11). SEM images confirmed the spherical structure. Panc-1 only spheroids had a more ellipsoidal structure than the spheroids grown with various ratios of stellate cells because the stellate cells acted as a support or scaffolding to promote a sphere-like shape. SEM analysis provided additional details on the cells’ arrangement inside the spheroids but did not allow any visual variations between the different spheroid compositions to be recorded. Additionally, real-time, optical microscopic BF imaging (Figs. 3B and S2A) allowed for daily evaluations of the spheroids’ formation parameters as a function of the variations in co-culture ratio.

Spheroid size as a function of incubation time (Fig. 3C) was also evaluated by BF imaging. The results showed different phases of formation: from day 1 to day 3, the cells were sedimented at the bottom of the well and began to organize themselves into spheres. Between days 3–7, the spheroid sizes were stable, but densification was observed, as shown by the optical density evaluation (end point: day 7). After 5 days, the sizes were recorded to be 974 μm ± 50, 952 μm ± 64, 770 μm ± 49, and 774 μm ± 87 for Panc-1, Panc-1/PSCs ratio 5-1, Panc-1/PSCs ratio 1-1, and Panc-1/PSCs ratio 1-2, respectively. Optical density (Fig. 3D) also indicated a significant variation in the formation and densification processes after day 5, especially for the 1-2 ratio (Panc-1 as a control for the significance study).

Viability testing with calcein AM and propidium iodide (Figs. 3B and S2B) indicated that all the spheres were viable and contained proliferative cells, especially those located in the outer layer (green fluorescence related to the calcein AM). Necrosis (red fluorescence) was mainly observed in the inside of the spheres. However, the necrosis/red fluorescence signal was more dispersed and sporadic in Panc-1 and Panc-1-PSCs ratio 5-1 than in Panc-1-PSCs ratios 1-1 and 1-2, indicating higher necrosis, most likely caused by the higher hypoxic effect that is managed by PSCs and hypothetically resultant from the presence of ECM. Analyzing the size, shape, and morphology of formation revealed the ability of the stellate cells to support the formation of the sphere by “scaffolding” its growth. These results indicate that the model, cell lines, and technique that we developed to obtained 3D cancer model spheroids confirmed observations from previous papers, especially in correlation with the production of a dense and rich ECM^8,22^.

### Nanoparticle interaction observed via fluorescence and PA analysis

**Figure 4:**
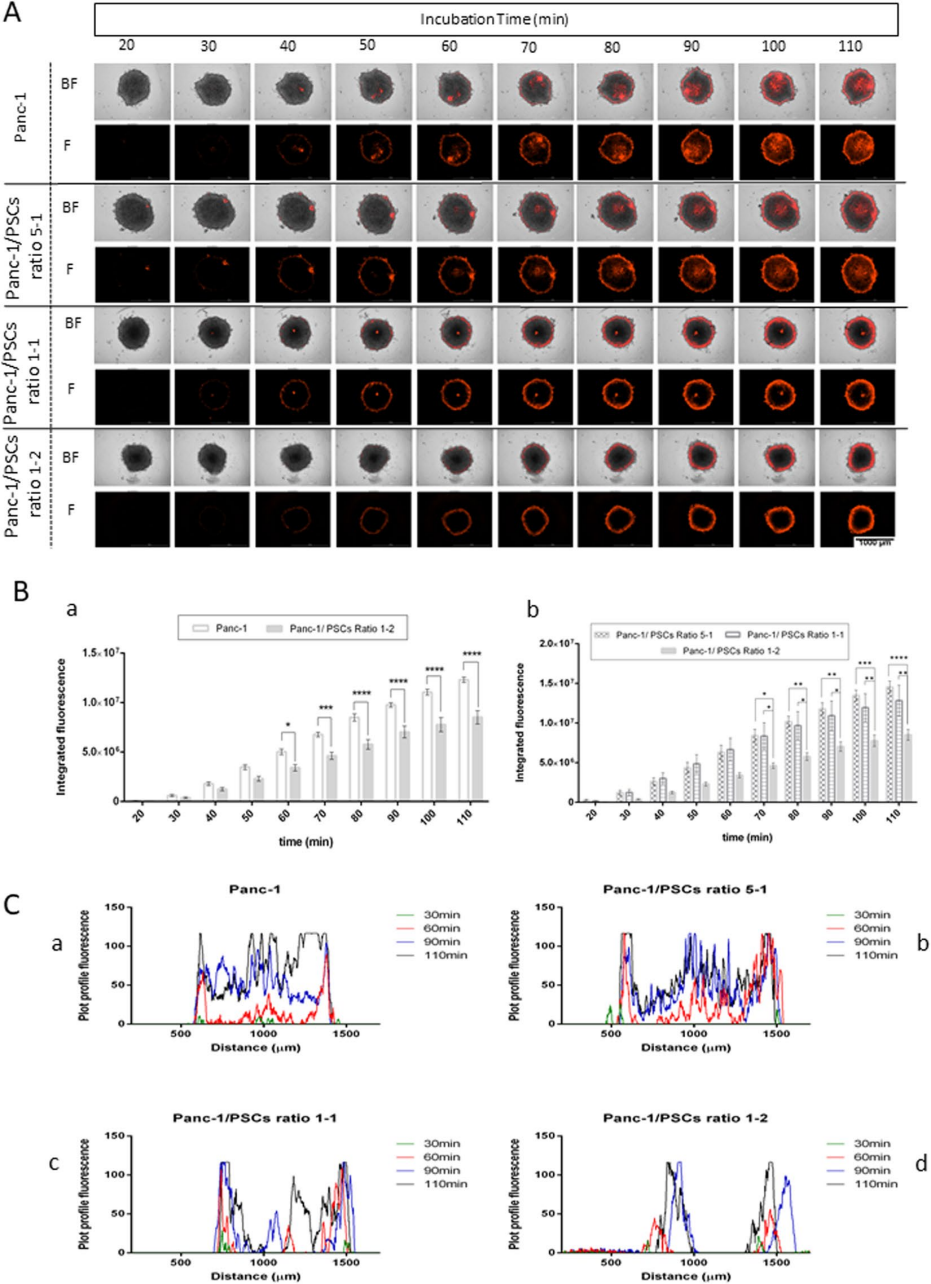
Real-time tracking and kinetic monitoring of the fluorescence on whole spheroids after AuNR-S-PEG-CO-NH-TR-BDP incubation. (**A**) BF and fluorescence (F) images of the different spheroids (Panc-1, Panc-1/PSC ratio 5-1, Panc-1/PSC ratio 1-1, Panc-1/PSC ratio 1-2) incubated with 50 μg.ml^−1^ of AuNR-S-PEG-CO-NH-BDP-TR, from 20 min to 110 min, captured every 10 min. Images recorded with BioTek Cytation 5. (**B**) (a) Evolution of the integrated fluorescence in Panc-1 and Panc-1/PSC ratio 1-2 in function of incubation time, with significance on the mean of the standard error with Panc-1 as a control; p ≤ 0.05, determined by two-way ANOVA using Sidak correction (n = 4). (b) Evolution of the integrated fluorescence in Panc-1/PSC ratios 5-1, 1-1, and 1-2 in function of incubation time, with significance on the mean of the standard error, with Panc-1/PSC ratio 5-1 as a control, with p ≤ 0.05, determined by two-way ANOVA using Sidak correction (n = 4). (**C**) Representative linear surface plotting based on the mean grey fluorescence plot profile at different times: 30, 60, 90, and 110 min with (a) Panc-1, (b) Panc-1/PSC ratio 5-1, (c) Panc-1/PSC ratio 1-1, and (d) Panc-1/PSC ratio 1-2.

**Figure 5:**
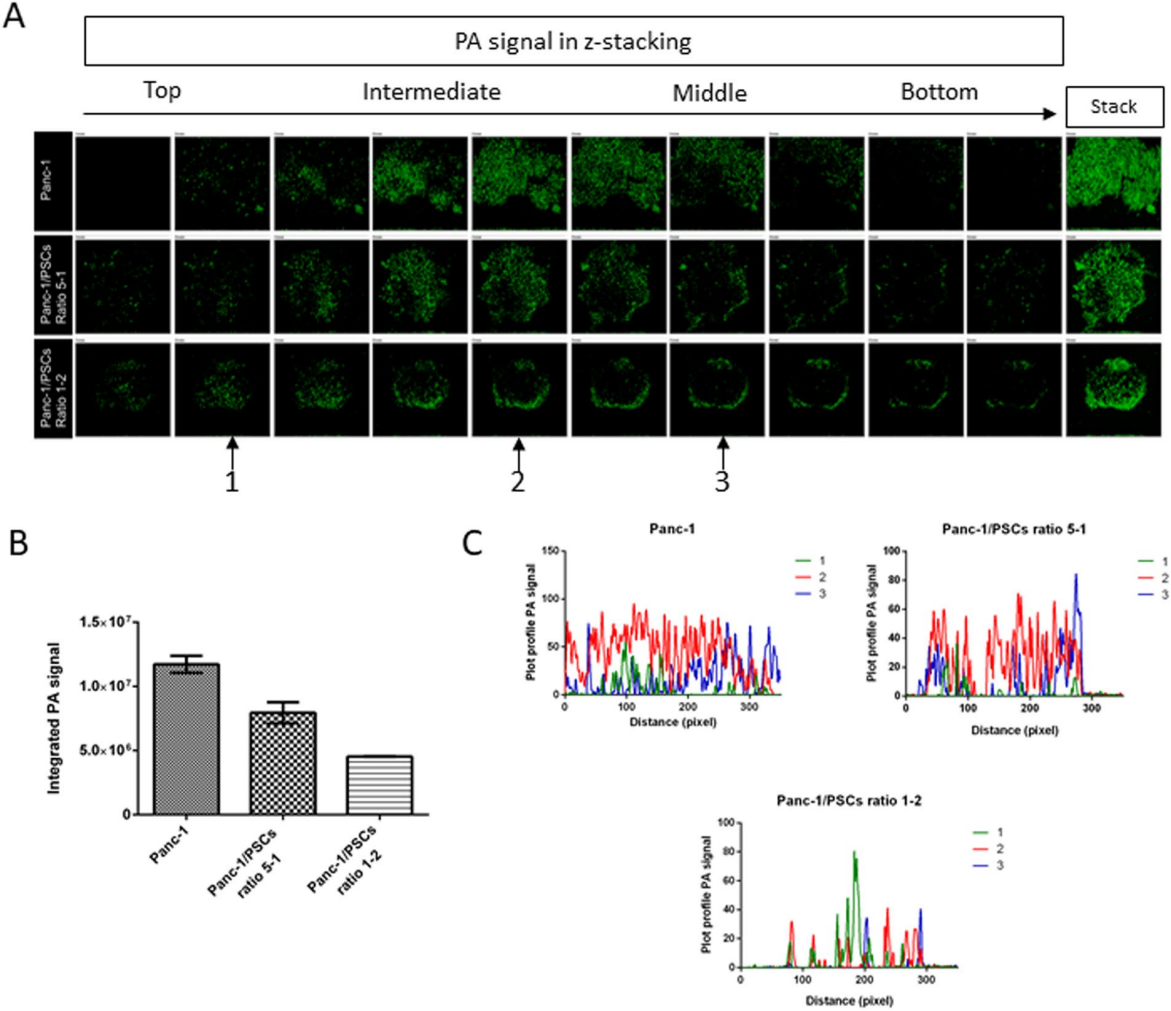
PA imaging on the whole spheroid, recorded by virtual z-stacking after incubation with AuNR-S-PEG-CO-NH-TR-BDP for 2 hours. (**A**) PA imaging in virtual z-stack (z = 10 μm) of the different spheroids (Panc-1, Panc-1/PSC ratio 5-1, Panc-1/PSC ratio 1-2) incubated with 50 μg.ml^−1^ of AuNR-PEG-CO-NH-BDP-TR for 2 hours. The arrows numbered 1, 2, and 3 at the bottom of the figure indicate the stack pictures used in C to plot the PA signal profile. (**B**) Evolution of the AuNR-PA signal accumulation recorded in each sphere (n = 2). (**C**) Line profile of PA signal through stack images 1, 2, and 3 for Panc-1, Panc-1/PSC ratio 5-1, and Panc-1/PSC ratio 1-2.

Performance, toxicity, and interaction of gold nanorods with various functionalizations with cancer cells have been investigated previously^47,48^. In this study, we used a 50-μg/mL concentration of conjugated nanoparticles for all the spheroid studies because it allows excellent imaging and no toxicity has been detected at that concentration. We focused on two major analyses: fluorescence only (Fig. 4A–C) and photoacoustic signature determination (Fig. 5A–C). Real-time monitoring of fluorescence-conjugated NP incubation was conducted using BioTek Cytation 5. The instrument was set at 37 °C to maintain normal biological culture conditions. To avoid fluorescence from the background of the media containing dispersed nanoparticles, the instrument was thresholded according to the sample at 20 minutes and according to the control spheroid (spheroid incubated in media without NPs). After 30 minutes, fluorescence was detected in all spheroids, as shown in the BF + F (bright field + fluorescence) and F (fluorescence) images (Fig. 4A,B). Comparison between Panc-1 and Panc-1/PSC ratio 1-2 (Fig. 4B) showed a significant variation in integrated fluorescence after 60 minutes of incubation. This variation was significantly higher in Panc-1 than in Panc-1/PSC ratio 1-2—reaching 1.2-fold higher after 110 minutes.

Comparison between the stromal-addition spheres in different ratios, with Panc-1/PSC ratio 5-1 as a control, indicated a significant variation of fluorescence after 70 minutes of incubation; ratio 5-1 was 1.2-fold and 1.7-fold higher than ratio 1-1 and ratio 1-2, respectively. Repartition of the fluorescence was also impacted by the ratio of stromal cells. The representative line profile plot of the fluorescence at different times (30, 60, 90, and 110 min) indicated a variation of fluorescence repartition inside the spheres. For Panc-1 and Panc-1/PSC ratio 5-1, the fluorescence increased uniformly in all the spheres as a function of time, but for ratios 1-1 and 1-2, the fluorescence was delayed and remained localized mainly within the outer layers of the spheroids (Fig. 4C). Generally, especially in the case of kinetic fluorescence imaging, spheroid images record non-adherent and floating systems. Consequently, light axial rotation of the sphere can be seen during the measurements, meaning that the images might not look at the same position. However, the measurement of the fluorescence profile was always recorded on the same spheroid position, time point after time point. Based on these results, we concluded that the fluorescence within each sphere is variable, leading us to hypothesize that the concentration and repartition of nanoparticles in each sphere could also be different. These results are in good correlation with published reports, which, by fluorescence tracking of fluorescent-tagged nanoparticles, found that rich stromal spheroids allow limited penetration due to the higher amount of ECM components, which hamper and shield the extracellular flow of nanostructures (in an avascular system)^25,38^.

To verify this hypothesis, we developed a photoacoustic analytic method and performed it on each whole spheroid (Fig. 5). PA data were collected from washed and fixed spheroids previously incubated with 50 μg/mL of conjugated AuNRs, immersed in DI water in a petri dish. Each condition was tested twice. Z-stack depth imaging ranging from 10–20 μm from the top to the bottom of the spheroids was performed and the images analyzed. The results were in good agreement with the previous fluorescence data. A stack-by-stack comparison of images within the same spheroid also showed variation in the repartition of the PA signal: Panc-1 and Panc-1/PSC ratio 5-1 possessed higher distribution, even in deeper depths inside the spheroids, while the Panc-1/PSC ratio 1-2’s signal was mainly concentrated in the outer layers (Fig. 5C). These results are similar to those recorded for the fluorescence plot profiles.

Therefore, a combinatorial approach of Cytation 5 and PA microscopy on the whole spheres allowed us to rapidly (instant imaging) and accurately visualize the distribution of the nanoparticles and the fluorescence of the corresponding dye used as a release model. We observed the ability of the nanoparticles to release the fluorescent dye in acidic conditions, and the PA technique determined the distribution of the nanoparticles within the spheroids based on the unique PA signature of the nanorods.

### Slicing of the spheroids and combined fluorescence-PTM analysis

In order to confirm the details of nanoparticle distribution within the spheroid, we utilized high-resolution photothermal microscopy (PTM) that is similar to the PA method utilized to observe the absorption contrast of the NPs but has much higher sensitivity and resolution. Indeed, data recorded in previous studies^53,54^ indicated that PTM offers absorption sensitivity ≥30–50- fold than PA *in vitro* and does not require an acoustic contact between the sample and transducer. Additionally, compared to the PA microscope, the PTM was built with a fluorescence microscope addition, providing the perfect approach to compare the signals of both techniques. However, our PTM can scan only ~47 × 47μm squares and requires stitching to recompose the images, which takes a longer time than PA data recording on a large sample (entire spheres). Advanced care needs to be given to the process of stitching in order to make sure that the data is accurately plotted.

**Figure 6:**
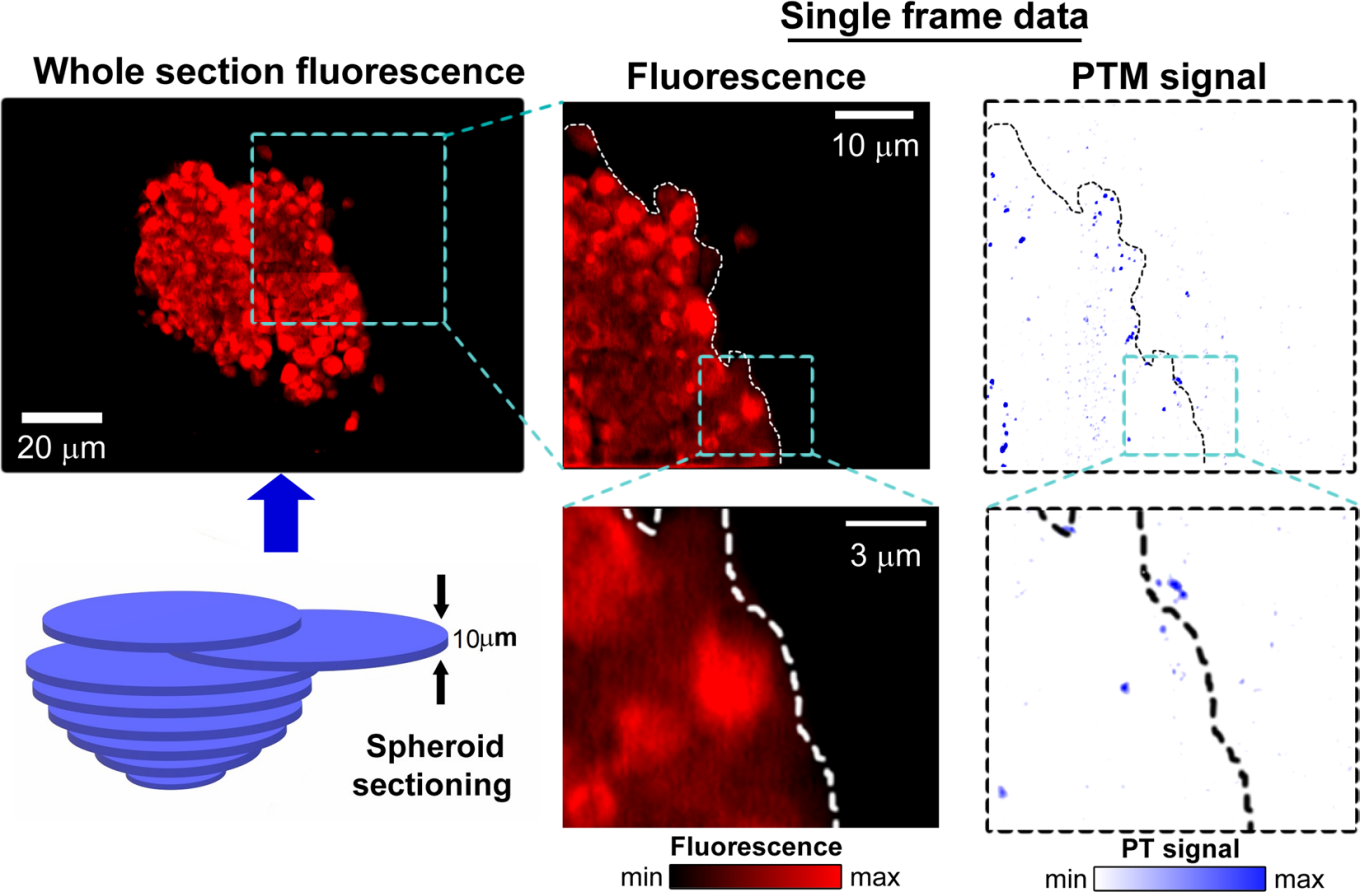
Principles of fluorescence and PTM analysis of spheroid sections. Blue dash line indicates the correlation between zoomed sections and the whole slice. White and black dash lines highlight the edge of tissues on the section.

In identical conditions as used in the previous experiments, spheroids grown for 5 days were incubated for 2 and 24 hours with 50 μg/mL of conjugated AuNRs. At both time points, they were washed and fixed with paraformaldehyde. They were then sliced at 10μm thickness. Instrumental microscopy, along with photothermal and confocal fluorescence detection, was used on selected slices to accurately locate the top, intermediate, and middle of each spheroid. Figure 6 shows the general principles of spheroid slicing, fluorescence/PTM quantification of the released dye (fluorescence), and NP location (PTM signal) (Fig. 6).

After 2 hours of incubation, fluorescence analysis on the slices showed that in Panc-1, Panc-1/PSC ratio 1-5, and Panc-1/PSC ratio 1-2 spheres, the nanosystems did not seem to have reached the core of the spheroids. The fluorescence analysis of the percentage of the area covered by the fluorescent dye inside the different stacks indicated more than 90% coverage for all the spheres but dropped to 74% at 10-μm depth for Panc-1/PSC ratio 1-2. In intermediate and middle slices for all spheres, fluorescence covering dropped below 40%.

**Figure 7:**
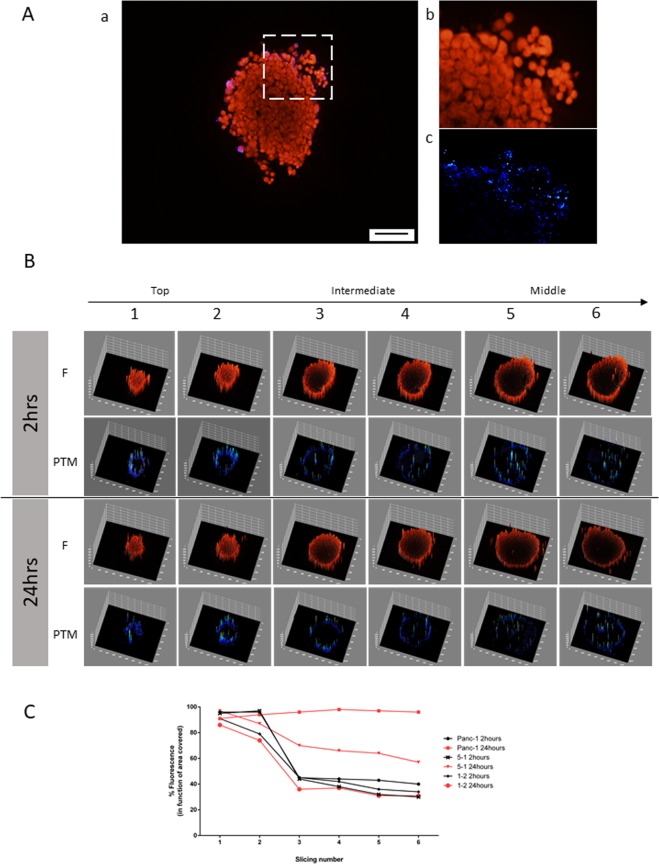
Fluorescence and PTM analysis on sections of the different spheroids after 2 and 24 hours of incubation with 50 μg.ml^−1^ of AuNR-S-PEG-CO-NH-BDP-TR: (**A**) Fluorescence (red) and PTM (blue) image recorded for slice 1 (representing the top of the spheroid), after 2-hr NP incubation, with (a) PTM and fluorescence images overlapping on the whole slice (scale: 100 μm), (b) fluorescence image of the white rectangle section from (a–c) PTM image of the white section from (a); (**B**) 3D representations of the fluorescence (F) and PTM images of two slices on the top, intermediate, and middle of Panc-1/PSC ratio 1-2 after incubation. (**C**) Evolution of percentage of fluorescence (as % of area covered) in each section in function of incubation time.

Extremely variable results were recorded after 24 hours of incubation: the fluorescent NP coverage of the intermediate and middle slices was ~97%, ~65%, and 30% for Panc-1, Panc-1/PSC ratio 5-1, and Panc-1/PSC ratio 1-2, respectively. Between 2 and 24 hours, the fluorescence in the core of Panc-1/PSC ratio 1-2 was unchanged. Also, compared to the analysis performed previously on the whole sphere, a small variation was visible between Panc-1 and Panc-1/PSC ratio 5-1, with 30% variation of cover in the deeper layer. PTM images confirmed the presence of NPs and indicated a variation of repartitions in function with the stroma present in the spheroids. Surprisingly, the relative number of NPs, especially within the outer layers of the spheroids, did not show any higher variation between 2 and 24 hours. The only significant variation between 2 and 24 hours was the ability of nanoparticles to flow deeper inside the spheroids, with the following trend: Panc-1 > Panc-1/PSC ratio 5-1 > Panc-1/PSC ratio 1-2. (Figs. 7, S3, and S4). However, it must be pointed out that the fluorescent signals and the NP signals did not always overlap (Fig. S5-video), especially after 2 hours of incubation. Our observations showed that the nanoparticles are processed by the cells, and the fluorescence dye could be released inside the spheroid environment.

As a general remark, we made sure that all the spheroids maintained their integrity during formation and NP incubation. If cracks or surface irregularity are visible, they are from the successive washing and handling, which also indicates that some spheroids, especially Panc-1, are more brittle than those containing stromal cells.

## Discussion

We have successfully developed an avascular, complex, co-culture 3D spheroid model made of human pancreatic stromal cells of different sizes and densities that can be used to study the interaction, penetration, and diffusion of loaded gold nanoparticles. We have demonstrated that these AuNRs enable a multiple-source tracking/imaging system—fluorescence, photoacoustic, and photothermal microscopy. Through these tracking/imaging methods, we confirmed that stromal cells in this 3D spheroid model act as a physical and/or chemical barrier for gold nanoparticle penetration. We analyzed the massive spheroids (dimension >500 μm) using a functionalized tracker and the inherent properties of the nanostructure. PTM and PA analysis highlighted the low penetration of the nanoparticles inside the 3D spheroid system.

It is important to note that the AuNRs were found to agglomerate on the outer layer of the 3D spheroids, as identified by their massive PA and PTM signals; these results were influenced by the spheroids’ stromal cell content. Our hypothesis is that the first layers of cells were quickly saturated, decreasing their ability to uptake more nanoparticles. At the same time, increasing incubation time allowed nanoparticles to penetrate closer to the core, especially if the stroma was not present. Recent studies suggest that endocytosis and interstitial cellular gaps regulate nanoparticle transportation within pancreatic 3D cancer models^52^.

Interestingly, the fluorescent and the nanoparticle (PTM images) signals did not always overlap (Fig. S5-video), even as early as after 2 hours of incubation. We observed that the NPs are processed by the spheroids, and the fluorescent dye could be released inside the corresponding environment. We also proved that fluorescence tracking to determine NP penetration is dependant of the chemical conjugation between the dye and the nanoparticles; for example, the EDC/NHS reaction and its ultra-labile amide bonding can enable a pH-triggered release.

Analysis and tracking of both the nanoparticles and the fluorescence are necessary in order to accurately determine the efficiency of the release model. In Panc-1 model, due to morphological specificity, both fluorescence and nanoparticles can flow closer to the core, which was not observed for the Panc-1/PSC ratio 1-2 system, possibly due to more corresponding necrosis and ECM, reducing the interstitial space. In consequence, the fluorescent dye—and, by extrapolation, a nanosystem—can have more difficulty being delivered.

This study indicates the need for further investigation of active targeting nanostructures, not only for cancer cells, but also for cancer stroma, and their action on its non-cellular components such as ECM (regulation, degradation), to overcome past failed therapies^5,19,55^.

## Methods

### AuNR synthesis and conjugation with NH_2_-TR-BDP fluorescent dye by EDC/NHS reaction

AuNRs were prepared using previous reported protocols^45,47,49^. First, AuNRs (1 batch of ~1,000 µg) were prepared from a seed solution. Briefly, the seed solution was prepared by stirring 5 ml of CTAB solution (0.2 M) with 5 ml of HAuCl_4_ (0.0005 M) and 600 µl of NaBH_4_ (0.01 M). Then, to synthesize AuNRs with an aspect ratio of ~ 3, 5 ml of CTAB (0.2 M), 150 µl of silver nitrate aqueous solution (0.004 M), and 5 ml of HAuCl_4_ (0.001 M) were combined. Next, 70 µl of ascorbic acid (0.0788 M) and 12 µl of seed solution were added, and the solution was kept at 30 °C for 40 minutes. The AuNRs were then purified twice at 10,000 rpm for 20 minutes and re-dispersed in DI (deionized) water. Second, AuNRs were covered with an HS-PEG-COOH solution. Briefly, the gold nanorods were dispersed in a 2-ml solution of carboxyl thiolated polyethylene glycol (HS-PEG-COOH) (M_W_ ~3,400), prepared by dissolving 2 mg/mL of PEG powder in 2 mM of NaCl solution, and maintained at 5 °C for at least 12 hours. The AuNR-S-PEG-COOH were then purified twice at 10,000 rpm for 20 minutes. Finally, the AuNR-S-PEG-COOH were dispersed in 1 mL of DI water for storing or in 1xPBS for immediate conjugation. An EDC/NHS reaction^42^ was used to conjugate the functionalized PEG‐coated nanorods with the fluorescent dye NH_2_-TR-BDP. 1 mL of 1000-μg/ml AuNR-S-PEG-COOH was dispersed in 1x PBS, then 57 μL of 10-mg/mL EDC were added, which had been previously dissolved in 1x PBS. The solution was orbitally shaken for 10-15 minutes. Then, 28 μL of 10-mg/mL NHS in 1x PBS were added, and the solution was shaken again for 10 minutes. 400 μL of NH_2_-TR-BDP, previously dissolved at 100 μg in 400 μL, were added and orbitally shaken for 4 hours. The solution was centrifuged at 10,000 rpm for 20 minutes, washed 3 times, and re-dispersed in 1 mL of 1x PBS.

### pH-controlled fluorescence release

1 batch of functionalized AuNR-S-PEG-CO-NH-BDP-TR was incubated with 1 ml of 1x PBS buffer at pH = 7 as a control, and 1 batch was incubated with 1 mL of sodium acetate buffer at pH = 5.5. The AuNR-S-PEG-CO-NH-BDP-TR (pH = 7) and AuNR-S-PEG-CO-NH-BDP-TR (pH = 5.5) were sonicated with a sonicator probe for 15 seconds and incubated in the cell incubator/biological culture at 37 °C, 5%CO_2_. Next, the tubes were centrifuged at 10,000 rpm for 30 minutes, and 100 μL of supernatant was collected and introduced in a 96-well-plate. Fresh buffer was introduced in the tube, sonicated for 15 seconds by sonicator probe, then incubated as mentioned before. Fluorescent reading was done with the microplate reader in fluorescence mode (Synergy H1 from BioTek, Vinooshi, VT). The samples were triplicate (n = 3). After 5 days, the samples with a pH of 7 were centrifuged, and sodium acetate buffer (pH = 5.5) was added. The same protocol as before was used to control the fluorescence release.

### Cell culture, 3D formation, and characterization

Panc-1 cells were grown in T75 culture flasks with DMEM containing 10% fetal bovine serum and 1% penicillin/streptomycin. Human primary PSCs in T75-poly-L-lysine modified culture flasks were cultured in the medium provided by the manufacturer (Sciencell™). All the cells were maintained in an incubator at 37 °C in a 5% CO_2_ atmosphere and then sub-cultured by trypsinization every 3-5 days, in limited passages (8 for PSCs and 14 for Panc-1). The cell culture media used for the 3D culture was DMEM/F12 complemented with 10% fetal bovine serum and 1% penicillin/streptomycin. The cells were initially seeded at ~10,000 cells in a 96-well, ultra-low attachment plate. Cells were co-cultured at different ratios of Panc-1 to PSCs (1:1, 1:2, and 5:1). Spheroids of Panc-1 alone and/or PSCs alone were used as controls. Media was changed every 2 days. Spheroids were imaged with Cytation 5 from Biotech from day 1 to day 7. BF images were taken using a x10 objective lens. Optical density was evaluated using ImageJ software after correct calibration every day. For viability imaging, the spheroids were grown for 5 days, then the media was removed, and the spheroids were washed at least 2 times with 1x PBS, previously warmed at 37 °C. Then, 100 μL of a solution containing 1 µL of calcein AM, 1 µL of diluted PI, and 98 µl of PBS were added for each spheroid. The spheroids were incubated at 37 °C then imaged with Cytation 5 in fluorescence microscopy mode, using GFP (green fluorescence protein) and TR (Texas red) filters for fluorescence imaging. For SEM imaging, the spheroids were cultured for 5 days in ultra-low adhesion plates. They were washed twice with 1x PBS. Individual spheroids were then immersed in 3% glutaraldehyde in the 0.1-M cacodylate buffer at pH 7.2 and maintained at 4 °C for 24 hours maximum. After being washed twice with the cacodylate buffer and twice with DI water, the individual spheroids were immersed in a 2% buffered solution of osmium tetroxide and maintained at 4 °C for 24 hours maximum. The spheres were then dehydrated by a series of absolute ethanol washings. Finally, the ethanol was removed, replaced by HMDS solution, and left under the hood until complete evaporation. The spheroids were then analyzed using SEM.

### Nanoparticle interaction on the whole sphere

The fluorescence analysis of the whole spheroids was conducted on 5-day-grown spheroids. 50 μg/mL of the nanorod conjugate) was added to each sphere. The 96-well-plate was then placed inside the Cytation5, already maintained at 37 °C, and every 10 minutes, a BF image and a fluorescence image were recorded using an x10 objective lens. Tracking was conducted for 110 minutes. For PA imaging on the whole sphere at 2 hours and 24 hours, the spheres were grown for 5 days, and 50 μg/mL of nanorod conjugate were added to each sphere. At the determined times, the spheroids were washed twice with 1x PBS and fixed with 4% PFA for ~24 hours at 37 °C. A custom laser scanning PA microscope was coupled to an inverted Olympus IX81 microscope (Olympus, Inc. Center Valley, PA), as described before^44,46^. Briefly, a 532-nm laser beam (pulse repetition rate: 10 kHz) scanned the sample in an XY raster pattern using a pair of galvo mirrors (6215 H, Cambridge Technologies, Lexington, MA). The laser beam was focused by using 10× (UPlan, Olympus Inc.) or 2.3 (Thorlabs, Newport, NJ) objectives from the bottom of the sample. The focal area of the transducers defined the field of view—120 µm for the 20-MHz focused transducer (V316, 12 mm focal distance, Olympus-NDT Inc,) and 1.2 mm for the unfocused 3.5-MHz transducer (model 6528101, 4.5 mm diameter; Imasonic Inc., Besançon, France). DI water was placed in the dishes containing the spheroids to enable acoustic measurement. A computer with a high-speed digitizer (PCI-5124, 12-bit card, National Instruments, Austin, TX) was used to amplify and record the PA signals (5662B, Panametrics). A digital waveform generator (DG4062, Rigol, Beijing, China) enabled control over the mirrors and system synchronization. To perform wide-area imaging, a 1.2× objective and 3.5-MHz transducers were utilized in mosaic mode by shifting the position of the samples (0.65 mm step) via a mechanical stage (Proscan II, Prior Scientific, Inc. Rockland, MA). Z-stack was used to determine the signal through the spheroids. The PA signal of the whole sphere was reconstituted using stacking and 3D stack images via NIH ImageJ.

### Nanoparticle interaction on the sectioned sphere

After 5 days of formation and various incubation times with the nanoparticles, the spheroids were washed at least twice with 1x PBS and fixed by adding 4% PFA for at least 1 hour at room temperature. The spheroids were then washed at least twice and embedded in cold molded agarose-derived gel^56–58^. The solid block was maintained under ethanol atmosphere until it was embedded in paraffin for microtomic slicing at a thickness of ~10 μm. The PTM setup has been described in detail previously and is summarized here^43,59^. Briefly, PT microscopy was carried out on a custom-built platform based on an invert Olympus IX73 (Olympus America, Inc., Central Valley, PA) using a 3-wavelength RGB Combiner (RGB46HF, Thorlabs, Newton, NJ) to combine 488-nm (fluorescence excitation: IQ1C45 (488-60) laser diode, Power Technology, Little Rock, AR), 532-nm (PT pump: LabSpec 532 nm DPSS Laser. Laserglow Technologies, LLS series, Toronto, Canada) and 635-nm (PT probe: LP637 SM Fiber-Pigtailed Laser Diode, Thorlabs, Newton, NJ) laser beams into a single mode fiber (Fig. S5). High-resolution confocal fluorescence and PTM imaging were performed at the same time by guiding laser beams using galvo-mirrors (GVSM002, Thorlabs, Newton, NJ) across the sample. Probe beam intensity was collected via a 40x objective placed above the sample and measured by an amplified photodiode (PDA10A, Thorlabs, Newton, NJ). The PT signal was extracted from the probe beam intensity modulation using a digital lock-in amplifier (MFLI, 500 kHz, 60MSa/s, Zurich Instruments, Switzerland) and recorded by LabView software. Conventional fluorescent imaging was done with a CCD DP80 camera (Olympus America, Central Valley, PA). The sliced spheroids were measured slice-by-slice. The PTM images were taken from a 47 × 47 µm area. Each spheroid required several PTM images, which were then reconstructed into one image by stitching in Adobe Illustrator CC and overlapped with the fluorescence signal. Final image brightness/contrast was adjusted using Adobe Photoshop CC. The plot line and 3D profile were analyzed using ImageJ.

## Supplementary information

Supplemental Figures.

Figure S5 | Projection of stack of fluorescnce vs PTM.
