# Supplementary material for: Tracking Gold Nanorods’ Interaction with Large 3D Pancreatic-Stromal Tumor Spheroids by Multimodal Imaging: Fluorescence, Photoacoustic, and Photothermal Microscopies
Source: Sci Rep. 2020 Feb 25;10:3362. doi: 10.1038/s41598-020-59226-6 (PMC7042370; doi:10.1038/s41598-020-59226-6)
Supplement: Supplementary file 1 — Supplemental Figures. [file 41598_2020_59226_MOESM1_ESM.docx]

Supporting Information

**Tracking Gold Nanorods’ Interaction with Large 3D Pancreatic-Stromal Tumor Spheroids by Multimodal Imaging: Fluorescence, Photoacoustic, and Photothermal Microscopies**

Emilie Darrigues ^1^, Zeid A. Nima ^1^, Dmitry A. Nedosekin ^2^, Fumiya Watanabe ^1^,
Karrer M. Alghazali ^1^, Vladimir P. Zharov ^2^, Alexandru S. Biris ^1^

^1^ Center for Integrative Nanotechnology Sciences, University of Arkansas at Little Rock, 2801 S University Avenue, Little Rock, AR, 72204, USA

^2^ Arkansas Nanomedicine Center, University of Arkansas for Medical Sciences, 4301 West Markham Street, Little Rock, AR 72205, USA

Correspondence and requests for materials should be addressed to E.D. (email: exdarrigues1@ualr.edu) or A.S.B (email: asbiris@ualr.edu)


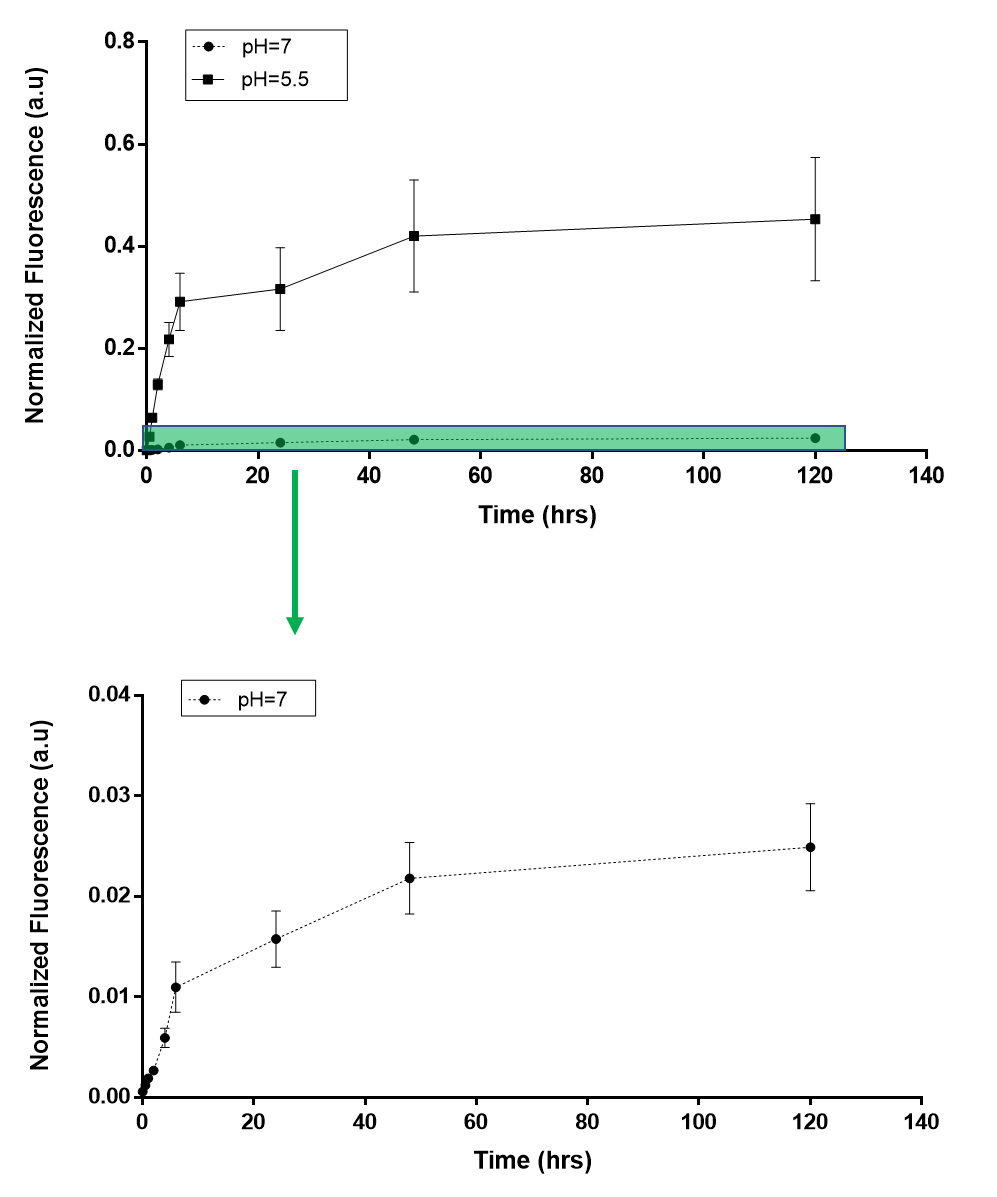


**Figure S1**: Normalized fluorescence release intensity of AuNR-S-PEG-CO-NH-TR-BDP incubated at 37°C at 5%CO_2_ in pH=7 for 30 min, 1hr, 2 hrs, 4 hrs, 6 hrs, 24 hrs, 48 hrs, and 5 days


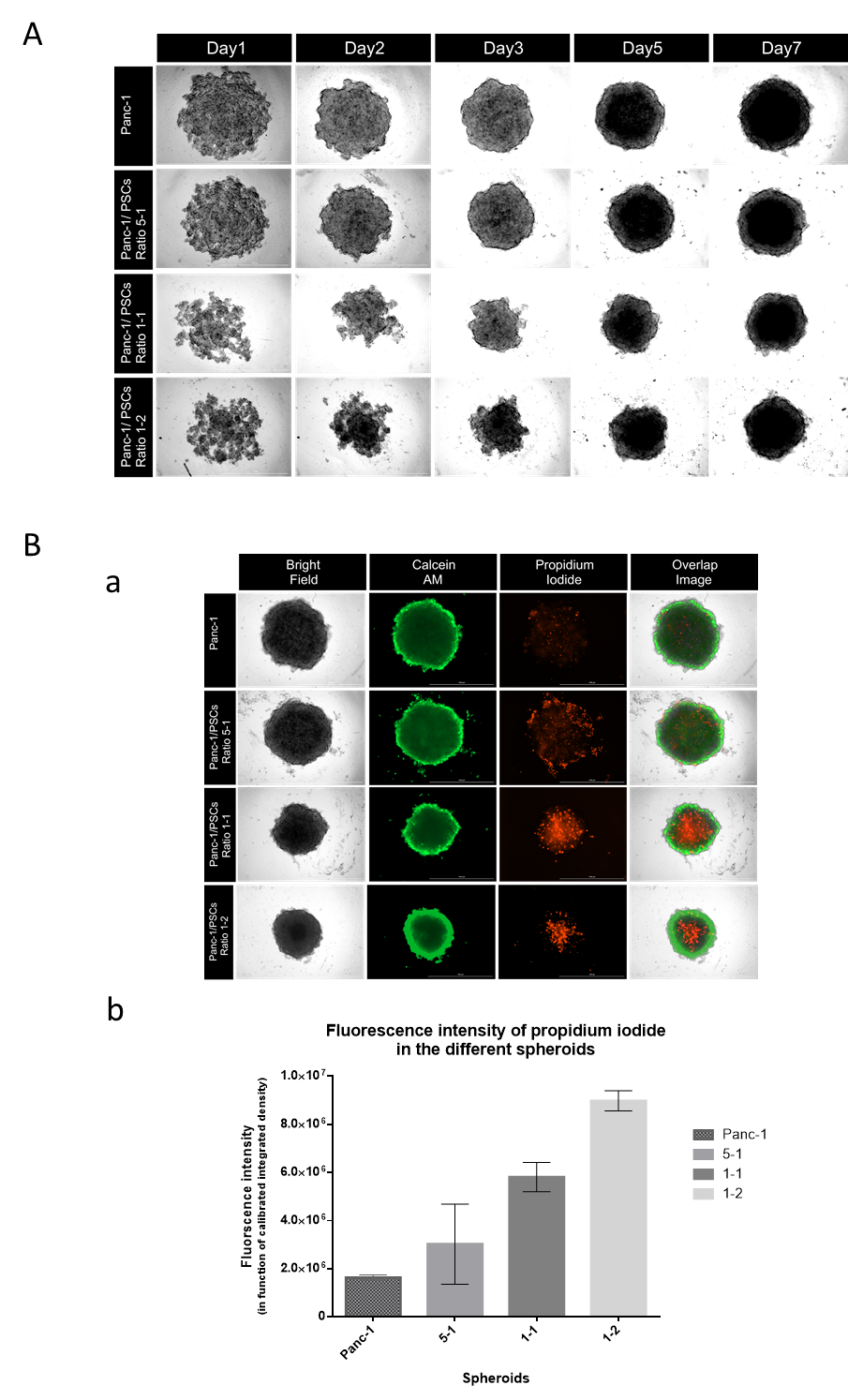


**Figure S2:** A) Optical microscope bright field images of the formation the different spheroids day-by-day, for day: 1, 2, 3, 5 and 7. B) Evaluation of necrotic/proliferative zones in the different 3D spheroids by using fluorescence microcopy with the viability assay Calcein Am (green)/ Propidium iodide (orange) with a) Bright field and fluorescent images and b) Fluorescence intensity based on the calibarted ntegrated density of Propidium iodide for the different spheroids (n=5).


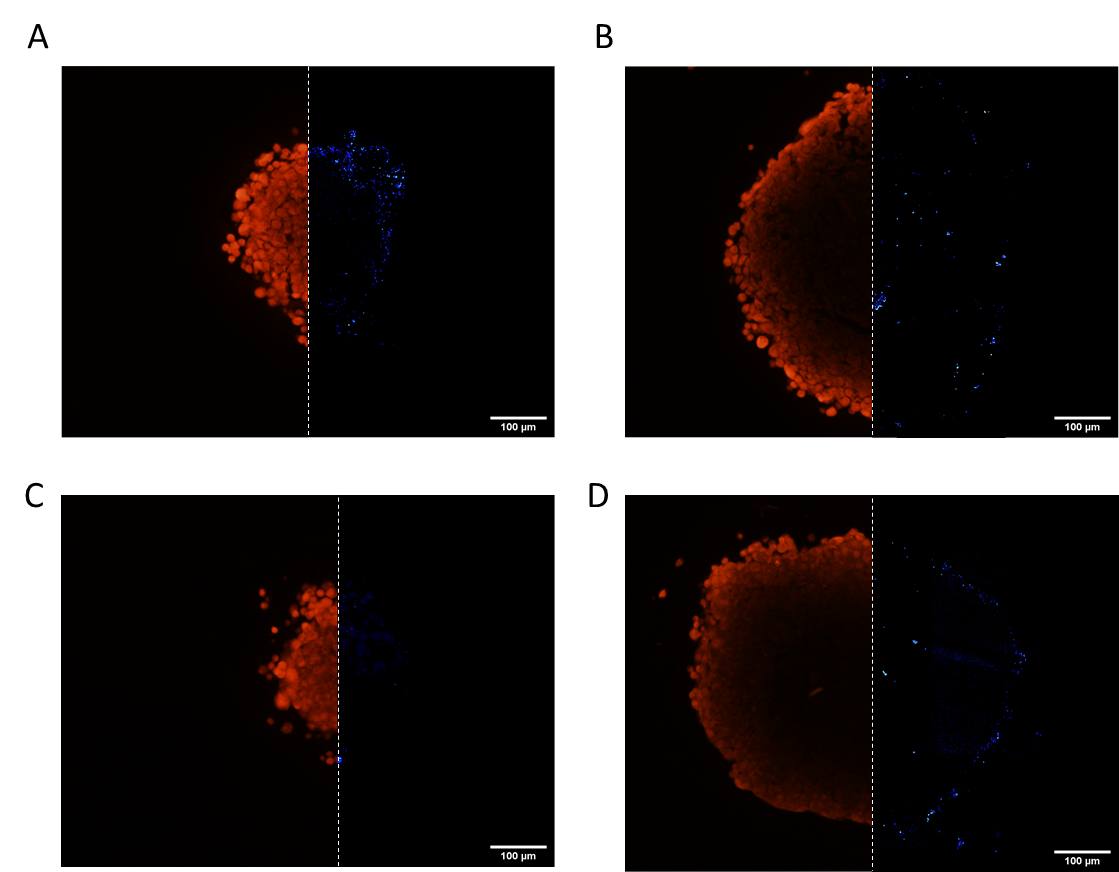


**Figure S3**: Imaging of Fluorescence (in red, left side) and PTM (in blue, right side) through z-stack slicing inside 3D culture for Panc-1/PSCs ratio 1: 2 with A) Slice 1 (top) and B slice 6 (middle) related to 2hours incubation with nanoparticles and C) Slice 1 (top) and D) Slice 6 (middle) related to 24hours incubation with nanoparticles


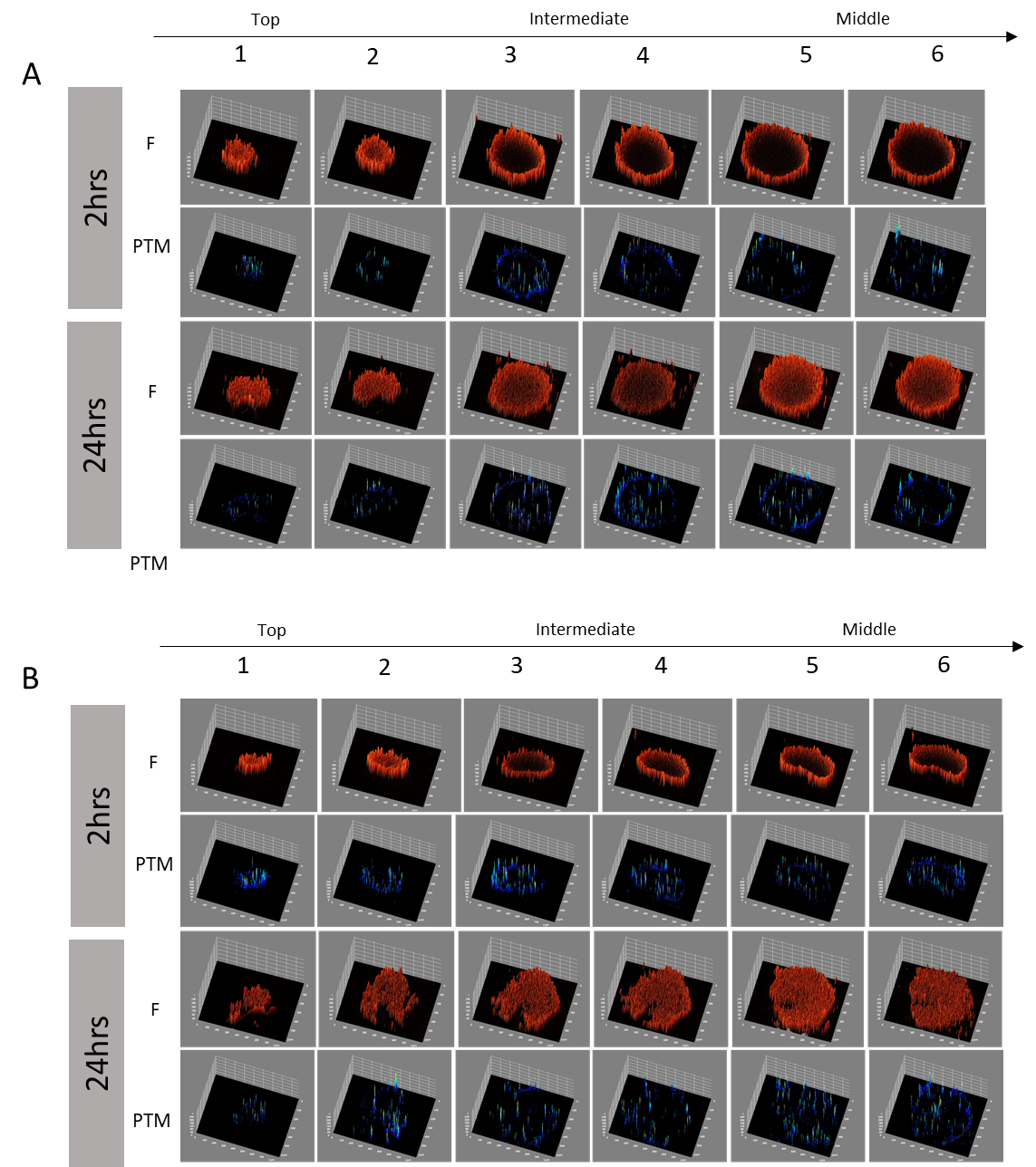


**Figure S4**: Imaging of Fluorescence and PTM through z-stack slicing inside 3D culture for A) Panc-1/PSCs ratio 5: 1 and B) Panc-1 alone

**Figure S5**: Video of a z-stack (5um) imaging PTM (bleue) and fluorescence (red) in the corner (47μmx47μm) of a 3D in spheroid Panc-1/PSCs 1:2 after 2hours incubation with nanoparticles


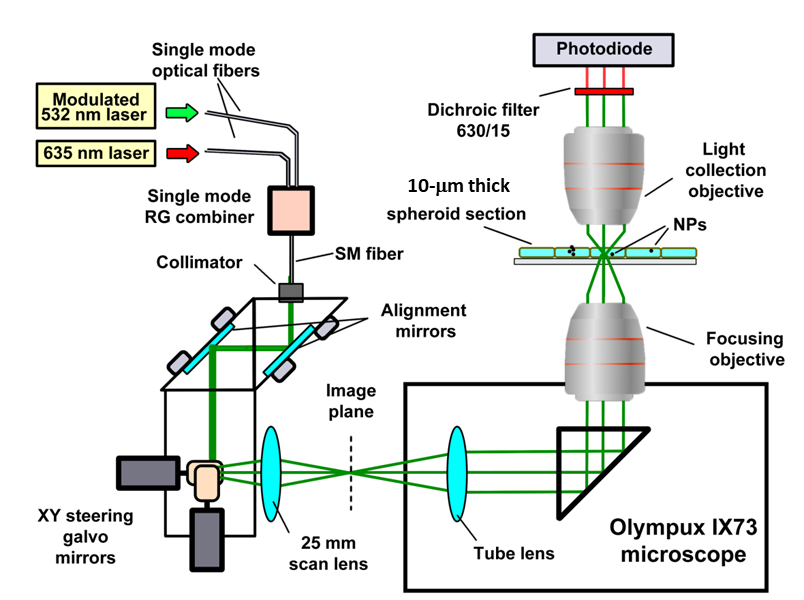


**Figure S6**: Schematics of PTM imaging system used for visualizing 10-µm thick spheroid sections.
